# Case report: Disseminated *Cladophialophora bantiana* phaeohyphomycosis in a dog with hepatic dysfunction, and concurrent ehrlichiosis

**DOI:** 10.3389/fvets.2024.1451299

**Published:** 2024-08-02

**Authors:** Flavio H. Alonso, Heather Fenton, Ananda Muller, Mark A. Freeman, Anne A. M. J. Becker, Kerry Rolph, Nicole Abramo, Gilda Rawlins, Liam Kitson, Erica Kessel, Mary Anna Thrall

**Affiliations:** ^1^Department of Biomedical Sciences, Ross University School of Veterinary Medicine, Basseterre, Saint Kitts and Nevis; ^2^Department of Comparative Medicine, Stanford University School of Medicine, Stanford, CA, United States; ^3^Australian Registry of Wildlife Health, Taronga Zoo, Mosman, NSW, Australia; ^4^Department of Clinical Sciences, Ross University School of Veterinary Medicine, Basseterre, Saint Kitts and Nevis

**Keywords:** fungal infection, hepatopathy, low-protein transudate, portal hypertension, acquired portosystemic shunt, ascites

## Abstract

A 1-year-old mixed breed dog initially presented with marked ascites due to a low-protein transudate resulting from portal hypertension. Laboratory evaluation revealed non-regenerative anemia, lymphopenia, thrombocytopenia, evidence of hepatic insufficiency [hypoalbuminemia, decreased urea, increased post-prandial bile acids, prolonged activated partial thromboplastin time (aPTT)] and *Ehrlichia canis* infection. Approximately a week later, the dog was declining and was euthanized. On autopsy, multifocal hepatic granulomas and acquired portosystemic shunts (APSS) were seen. Imprint cytology revealed fungal hyphae and pyogranulomatous inflammation in the liver and brain. Disseminated *Cladophialophora bantiana* phaeohyphomycosis was diagnosed by histologic examination, culture and PCR. Immunosuppression due to ehrlichiosis is suspected to have predisposed this animal to fungal infection. To the authors’ knowledge, this is the first report of *C. bantiana* in the West Indies.

## 1 Introduction

Phaeohyphomycosis refers to infections caused by a heterogenous group of saprophytic and dematiaceous fungi. *Cladophialophora bantiana* (synonyms for which include C. *bantianum*, *C. trichodes* and *Xylohypha bantiana*) is a member of the phylum *Ascomycota*, and is a mitosporic melanized mold which is a recognized cause of cutaneous and systemic disease in people (1). This organism demonstrates marked neurotropism, and therefore, systemic disease most commonly affects the central nervous system (CNS). A recent review article identified 120 case reports of C. *bantianum* infection in people (2). *C. bantiana* is part of the normal fungal biota on the hair of dogs (3), but cutaneous and systemic infections in dogs, cats, tigers, horses, and alpacas have been reported (4, 5). The following is a case report of a dog with ascites, *Ehrlichia canis* infection and disseminated phaeohyphomycosis that resulted in chronic liver disease, portal hypertension, acquired portosystemic shunts (APSS) and abdominal low-protein transudate formation. This case report was written in agreement with the most updated version of the CARE guidelines (6).

## 2 Case description

A 1-year-old, mixed breed, male neutered dog was presented to Ross University Veterinary Clinic, in St Kitts, West Indies with a several week history of abdominal distension, weight loss, and anorexia. Routine vaccinations and tick, flea, and heartworm preventatives had been given. On physical examination, the dog was bright and alert. Body condition score was 2 out of 9. Marked abdominal distention with a fluid thrill were noted. An initial neurological examination revealed no abnormalities.

Blood samples were collected on day of presentation (day 0) and at revisits on days 2 and 8 (Table 1) for complete blood counts (CBC, Vetscan HM5 hematology analyzer, Zoetis) and biochemical analyses (Vetscan VS2 Chemistry Analyzer, Zoetis). Hepatic dysfunction was evidenced by the combination of hypoalbuminemia, decreased urea, increased post-prandial bile acids, and prolonged partial thromboplastin time (aPTT). The non-regenerative anemia and thrombocytopenia were thought to be associated with ehrlichiosis, and the lymphopenia was likely due to stress, although immunosuppression could not be excluded. An immunochromatographic test (SNAP 4Dx Plus Test, IDEXX, United States) demonstrated a positive antibody test for *Ehrlichia canis*, *E. ewingii*, or a combination of the two. Due to antibody cross-reactivity, immunochromatography is not able to distinguish infections caused by these 2 pathogens.

**Table 1:** Complete blood counts, coagulation and serum biochemistry panels at different moments in a dog with disseminated *Cladophialophora bantiana* phaeohyphomycosis.

	Day 0	Day 2	Day 8	Reference interval	Units
Erythrocytes	**3.38**	**3.10**	**4.33**	5.5–8.5	×10^12^/L
Hemoglobin	**5.7**	**5.2**	**7.6**	12.0–18.0	g/dL
Hematocrit	**20.2**	**18.5**	**25.7**	37.0–55.0	%
MCV	60	60	**59**	60.0–77.0	fL
MCH	**16.8**	**16.8**	**17.4**	19.5–24.5	pg
MCHC	**28.2**	**28.3**	**29.5**	31.0–39.0	g/dL
Abs Retic	41	**62**	ND	0.0–60.0	×10^9^/L
RDW	18.9	18.9	18.2	14.0–20.0	%
Platelets	**12**	**32**	**126**	165–500	×10^9^/L
MPV	9.4	9.1	8.4	3.9–11.1	fL
Total leukocytes	**4.23**	**5.80**	**4.67**	6.0–17.0	×10^9^/L
Lymphocytes	**0.19**	**0.28**	**0.10**	1.0–4.8	×10^9^/L
Monocytes	0.33	0.32	**0.00**	0.2–1.5	×10^9^/L
Neutrophils	3.68	5.17	4.40	3.0–12.0	×10^9^/L
Eosinophils	0.04	0.04	0.00	0.0–0.8	×10^9^/L
PT	ND	ND	17.2	14–19.0	Seconds
aPTT	ND	ND	**136.6**	75–105.0	Seconds
BUN	10	**5**	**4**	7.0–25.0	mg/dL
Creatinine	0.6	0.5	0.4*	0.3–1.4	mg/dL
ALT	**147**	**278**	**242**	10–118	IU/L
ALP	**171**	**158**	**142**	20–150	IU/L
GGT	**9**	6	**9**	0–7	IU/L
tBil	0.3	0.3	0.3	0.1–0.6	mg/dL
Bile Acid	23	**40**	**26**	0.0–25	IU/L
Post prandial BA	ND	**46**	ND	00–25	IU/L
Total Protein	6.4	6.8	ND	5.4–8.2	g/dL
Albumin	**2.2**	**2.3**	**1.8**	2.5–4.4	g/dL
Globulin	4.2	4.5	ND	2.3–5.2	g/dL
Glucose	89	82	72*	60–110	mg/dL
Cholesterol	218	230	202	125–270	mg/dL
Amylase	1,067	1,130	ND	200–1,200	IU/L
Calcium	9.7	10.1	ND	8.6–11.8	mg/dL
Ionized calcium	ND	ND	1.4*	1.12–1.4	mmol/L
Phosphate	6	5.8	ND	2.9–6.6	mg/dL
Sodium	142	143	144*	138–160	mmol/L
Potassium	4.9	4.8	4.7*	3.7–5.8	mmol/L

Bold values are outside the reference interval.

Abs Retic, absolute reticulocytes; ALP, alkaline phosphatase, ALT, alanine aminotransferase; aPTT, activated partial thromboplastin time; BA, bile acids; BUN, blood urea nitrogen; GGT, gamma-glutamiltransferase; MCH, mean corpuscular hemoglobin; MCHC, mean corpuscular hemoglobin concentration; MCV, mean corpuscular volume; MPV, mean platelet volume; PT, prothrombin time; RDW, red blood cell distribution width; tBil, total bilirubin.

**Figure 1:**
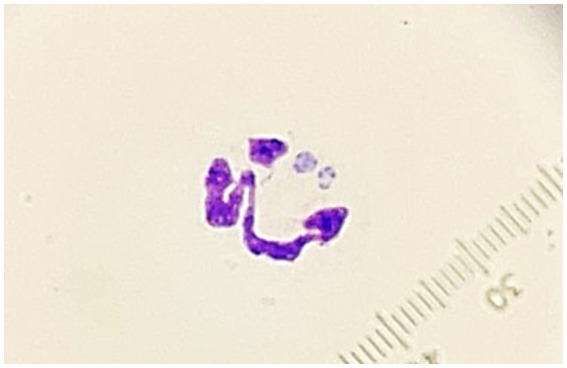
Photomicrograph of a neutrophil containing morulae observed in an abdominal fluid specimen from a dog with disseminated *Cladophialophora bantiana* phaeohyphomycosis and ehrlichiosis (1,000×, Wright’s Giemsa).

Thoracic and abdominal ultrasonographies were performed. The only abnormalities seen were an enlarged liver, and a large volume of hypoechoic fluid within the abdomen. Analysis of abdominal fluid revealed a low-protein transudate with a total nucleated cell count of 140 cells/μL (Vetscan HM5 hematology analyzer, Zoetis) and total protein estimate of 0.2 g/dL. Predominate cell types were macrophages and nondegenerate neutrophils. Some of the neutrophils contained intra-cytoplasmic inclusions interpreted as *Ehrlichia* spp. Morulae (Figure 1).

The dog was treated with Doxycycline for ehrlichiosis and received a single injection of Furosemide (1.5 mg/kg), followed by Spironolactone 1.1 mg/kg twice daily. The dog presented 6 days after initiation of therapy and the ascites had improved slightly. A second revisit appointment was planned, but the dog was not doing well, and the appointment was canceled. He began stumbling and falling and collapsed 5 days later and was humanely euthanized.

**Figure 2:**
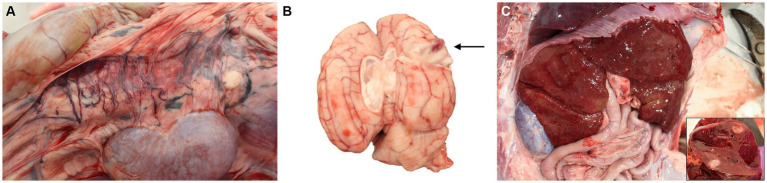
Photographs of post-mortem examination of a dog with disseminated *Cladophialophora bantiana* phaeohyphomycosis. **(A)** Multiple extrahepatic tortuous vessels are noted along the inferior vena cava within the mesentery, consistent with acquired portosystemic shunting. **(B)** A 1.5 cm in diameter, focal, slightly raised, soft white nodule with red center is observed within the mid-dorsal right parietal lobe of the cerebrum with a red center that extends deep within the cerebrum (arrow). **(C)** Gross photograph of the liver and of a cross-section of one of the hepatic nodules (inlet).

**Figure 3:**
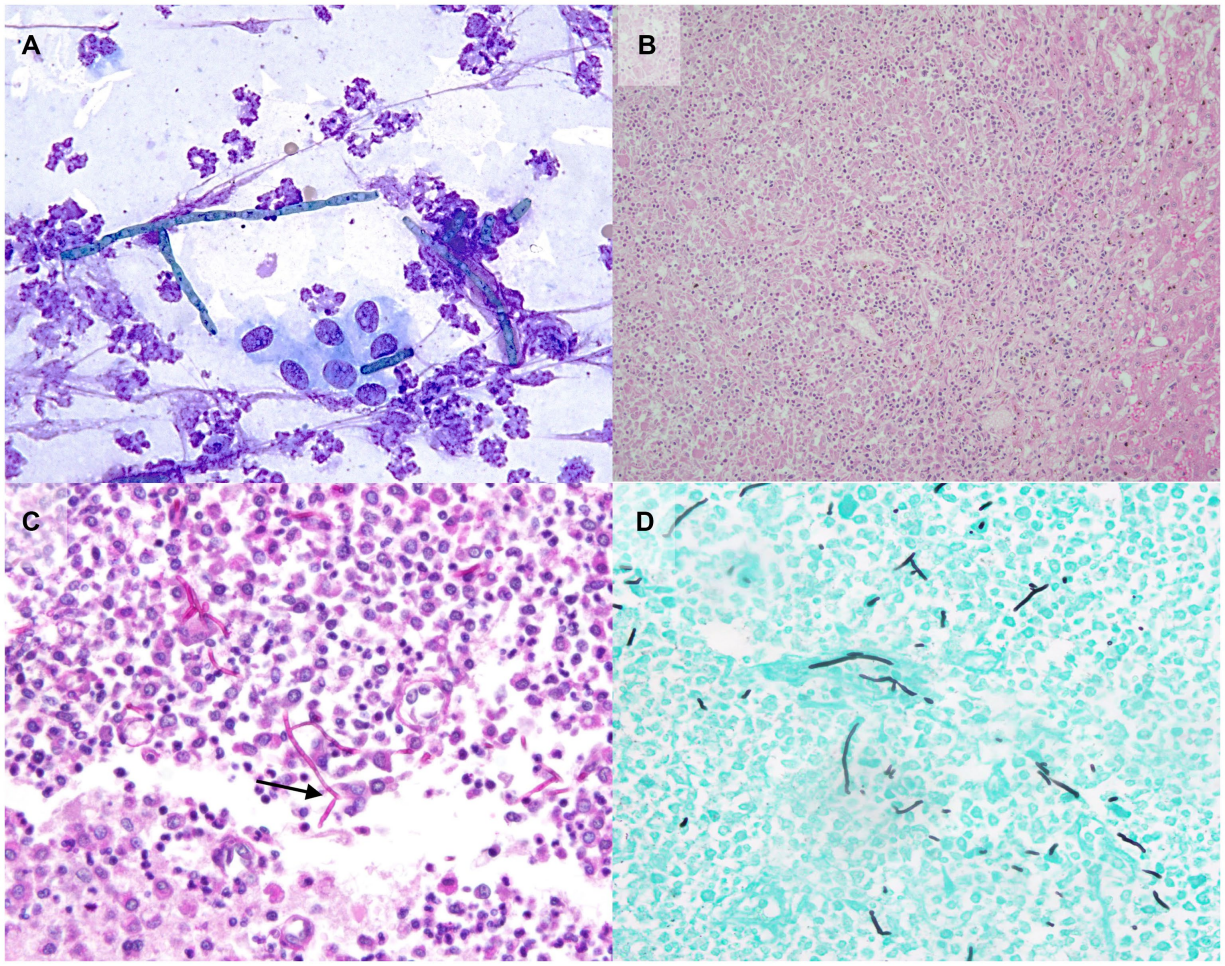
Photomicrographs of aspirate and tissue samples from a dog with disseminated *Cladophialophora bantiana* phaeohyphomycosis and ehrlichiosis. **(A)** Note the pyogranulomatous inflammation with fungal hyphae on the cytology of a liver nodule aspirate (500x, Wright’s Giemsa). Histopathology confirmed severe, multifocal to coalescing, chronic pyogranulomatous hepatitis (**B**, 200x). Special histochemical staining of a brain nodule with Periodic Acid-Schiff (**C**, 100x) and Grocott’s Methenamine Silver (D, 100x) is highlighting the hyphae which are approximately 5-9 μm in diameter, septate with rare 45-degree angle and-dichotomous branching (arrow).

Autopsy demonstrated a large volume of ascitic fluid with multiple extrahepatic tortuous vessels along the inferior vena cava within the mesentery medial to the kidneys, consistent with acquired portosystemic shunts (Figure 2). The liver and spleen were 3- to 4-fold enlarged, the kidneys were mildly enlarged, and the lymph nodes were variably firm and prominent. Multifocal nodules, that were white to tan, soft, and 2–15 mm in greatest diameter by 2–8 in smallest, were noted in liver, spleen, kidneys, myocardium and pancreas. The brain had a single similar nodule that was approximately 1.5 cm in diameter, located in the right mid-dorsal parietal lobe and contained central hemorrhage (Figure 2). Cytologic imprints showed pyogranulomatous inflammation and numerous fungal hyphae. Histopathology confirmed severe, multifocal to coalescing, chronic pyogranulomatous hepatitis, nephritis and adrenalitis with fungal sepsis and necrotizing meningoencephalitis (Figure 3). The liver also showed histologic evidence of diffuse rarefactive hepatocellular degeneration, extensive sinusoidal congestion, and multifocal to coalescing necrosis.

**Figure 4:**
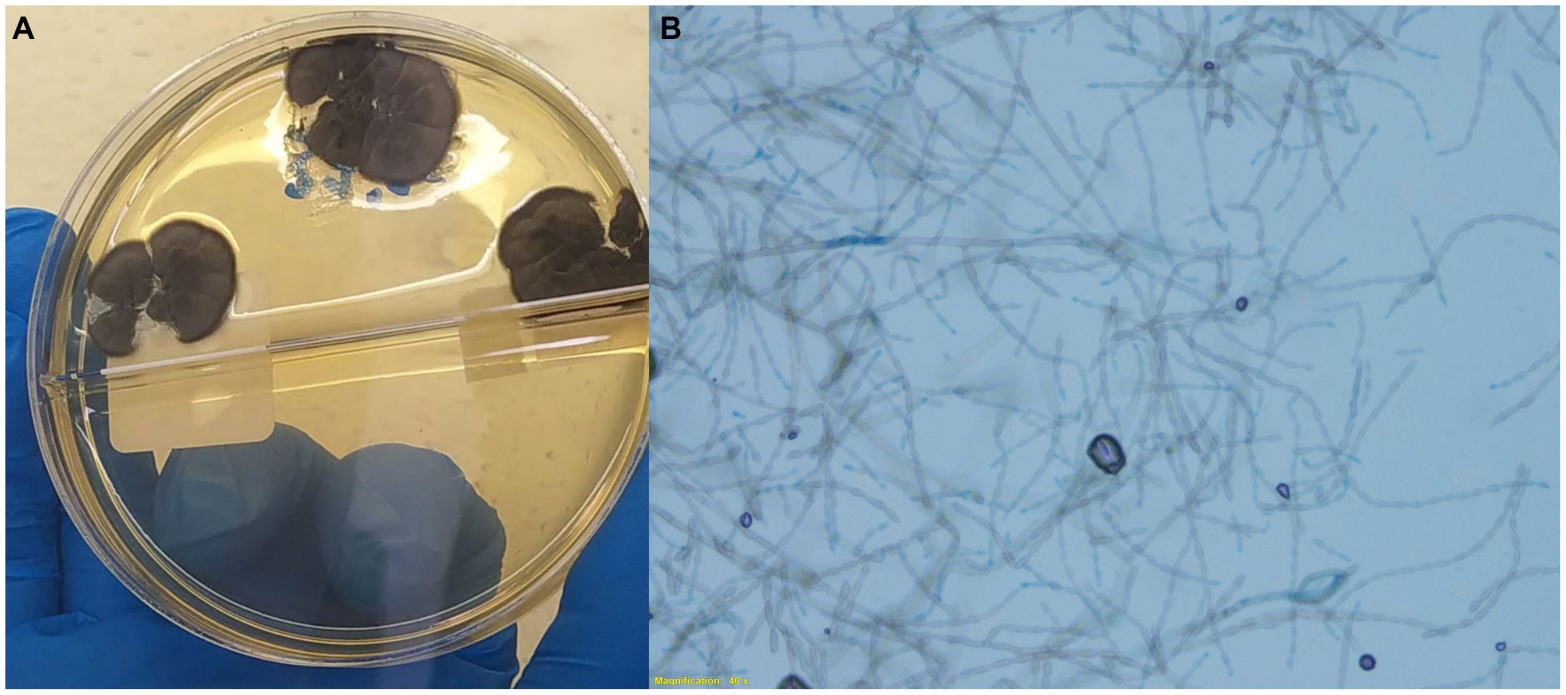
Culture of liver swab (Sabouraud dextrose agar medium) from a dog with disseminated phaeohyphomycosis. Note the downy texture and gray black colonies **(A)** with elliptical conidia in long, wavy, sparsely branched chains (**B**, wet mount, 400x). The species was confirmed as Cladophialophora bantiana with PCR and genome sequencing.

A fungus with dark gray, branched hyphae was recovered in culture (Sabouraud dextrose agar medium under 30°C) (Figure 4). The isolate along with fragments of brain were further analyzed by rRNA gene sequencing, with a nested PCR first amplifying the 3′ end of 18S rRNA gene to the ITS2 region and internal primers subsequently targeting the ITS1 region. Obtained sequences presented 99.8% similarity to *Cladophialophora bantiana* using BLAST searches in GenBank.

To further identify the rickettsial infection, DNA was extracted and purified from whole blood in EDTA and peritoneal fluid. Genomic DNA from blood and fluid were tested for 16S rRNA gene in *Anaplasmataceae* via conventional PCR (7); 16S rRNA gene for the sequencing *Anaplasmataceae* via conventional PCR (Size of product 866 bp); dsb gene for sequencing of *Ehrlichia* sp. (409 bp) (8) msp2 gene of *A. phagocytophilum* via conventional PCR; msp2 gene of *A. phagocytophilum* via real time PCR; p28 gene for the detection of *E. ewingi* via conventional PCR. Both blood and abdominal fluid were positive for a 16S screening, 16S and Dsb sequencing protocols. Obtained sequences (16S and Dsb) presented 98–99% identity to *E. canis*. Samples were negative for all the other tested genes and pathogens, excluding the possibility of co-infection with *A. phagocytophilum* and *E. ewingii*.

## 3 Discussion

This is, to the best of the authors’ knowledge, the first report of *C. bantiana* infection in any species within the West Indies. Canine phaeohyphomycosis caused by *C. bantiana* has rarely been reported. All reported cases in dogs had documented neurological signs, although lesions were noted in other organs in some cases (9–12). Another case of canine systemic phaeohyphomycosis caused by *Cladophialophora bantiana* that also affected the liver was recently reported (13). Information regarding the patient’s liver function, co-infection status or effusion formation was not provided.

Disseminated fungal disease is often associated with immunosuppressive pre-conditions (14). Many dogs with systemic mycosis have been treated with prolonged or high-dose immunosuppressive therapy such as glucocorticosteroids (15). However, over half of the reported human cases of *C. bantiana* infection were in immunocompetent individuals (2). In dogs, systemic infections have been reported in 14 dogs ranging from 4 months to 12 years of age. Of these, 8 had no known underlying disease. Lymphopenia, leukemia, distemper, diabetes mellitus, skin scratches and corticosteroid treatment were possible predisposing factors in some (4). Most of these were not present in this patient but, even though this patient was vaccinated, distemper could not be fully excluded, and lymphopenia was present at marked degree. Splenic lymphoid depletion found in this patient could represent further evidence of immunosuppression, as demonstrated in people with AIDS (16) and fowl under steroid therapy (17). One dog infected with *C. bantiana* was concurrently infected with *E. canis* (12), and it is possible that ehrlichiosis may have resulted in immunosuppression in this patient.

Even though the mechanisms are not yet completely defined, there seems to be a body of knowledge supporting the association between vector-borne diseases and immunosuppression. Human granulocytic anaplasmosis has been shown to be associated with evidence of immunosuppression, opportunistic infections and organ failures (18–22). Additionally, many dogs persistently infected with *E. canis* die of secondary infections during the pancytopenic or chronic phase of canine ehrlichiosis (23, 24). There have been a few reports of veterinary species and people with fungal infections suspected to be secondary to immunosuppression due to vector borne disease (25, 26). Results of a 3-year-long longitudinal study indicated that *E. canis* infection likely predisposed dogs to *Leishmania infantum* infection (27).

Structures consistent with morulae were found in neutrophils in the abdominal fluid sample of this patient (Figure 1), but not in peripheral blood. PCR analysis of the fluid was positive for *E. canis*. Even though this species is typically observed within monocytes, reports of dogs in which the inclusions were found within neutrophils of fluid samples including peritoneal fluid and cerebrospinal fluid have been documented (28, 29). There were insufficient erythrocytes in the abdominal fluid sample analyzed to suspect blood contamination or hemorrhage and we, therefore, consider possible that engulfment of the rickettsial organisms by alternative phagocytes might be more likely when infection extends to unconventional tissues, as seen in this case.

A limitation of this report is the fact that neither a bone marrow aspirate nor a core biopsy examination was performed and, therefore, myeloid hypoplasia could not be investigated. The patient was, however, pancytopenic, which could support a state of chronic, or at least persistent infection. We speculate that observation of morulae in an atypical sample (i.e., body fluid) could further support advance of *E. canis* infection to subsequent tissues, characterizing a more spread and established stage of the disease in this patient. It’s also possible that this patient was under another type of immunosuppressive condition, of either acquired or congenital origin, and persistent *E. canis* infection represented just another manifestation of that. Even though unlikely, there is still a chance that phaeohyphomycosis was primary (e.g., fungal omphalophlebitis, as stated above) and immunosuppression secondary.

Although fungal organisms and inflammation were present in multiple organs, the liver seemed most affected by dysfunction (i.e., hypoalbuminemia, low serum urea, increased post-prandial bile acids, prolonged aPTT, and mild microcytosis). The ascitic fluid was a low-protein transudate, caused by portal hypertension. Collateral neovascularization (i.e., shunt formation) has been described secondary to fungal infection in people (30). It is, however, unclear how often disseminated mycosis leads to hepatic failure. To the best of the authors’ knowledge, this is the first description of a case of systemic phaeohyphomycosis leading to multifocal and severe granulomatous hepatitis, hepatic dysfunction, portal hypertension, and ascites.

## 4 Conclusion

Because the fungal organisms were readily seen in postmortem cytology imprints, they would have likely been seen in an aspirate. Liver cytology or biopsy were not performed in this patient due to the thrombocytopenia and prolonged aPTT. While the thrombocytopenia was attributed to ehrichiosis, and the prolonged aPTT was attributed to liver dysfunction, disseminated intravascular coagulation (DIC) was also a possibility, but no additional tests to confirm or discount DIC were performed. In this case, a premortem diagnosis would not have likely influenced the outcome, since the disease was widespread including the brain, multiple acquired portosystemic shunts had developed, and the dog had ascites on presentation, which is a very poor prognostic sign in dogs with APSSs. The significance of morulae-like inclusions in neutrophils in the abdominal effusion is not known but might indicate chronic ehrlichiosis and related immunosuppression.

## Data availability statement

The original contributions presented in the study are included in the article/supplementary material, further inquiries can be directed to the corresponding author.

## Ethics statement

Ethical approval was not required for the studies involving animals in accordance with the local legislation and institutional requirements because this is a case report and, as such, it involved no experimentation on animals. Written informed consent was obtained from the owners for the participation of their animals in this study.

## Author contributions

FA: Conceptualization, Data curation, Investigation, Supervision, Writing – original draft, Writing – review & editing. HF: Writing – review & editing. AM: Writing – review & editing. MF: Writing – review & editing. AB: Writing – review & editing. KR: Writing – review & editing. NA: Writing – review & editing. GR: Writing – review & editing. LK: Writing – review & editing. EK: Writing – review & editing. MT: Writing – review & editing.
